# Minimally Invasive Platforms in Biosensing

**DOI:** 10.3389/fbioe.2020.00894

**Published:** 2020-08-31

**Authors:** Prem C. Pandey, Govind Pandey, Roger J. Narayan

**Affiliations:** ^1^Department of Chemistry, Indian Institute of Technology (BHU), Varanasi, India; ^2^Department of Pediatrics, King George Medical University, Lucknow, India; ^3^Joint Department of Biomedical Engineering, North Carolina State University, Raleigh, NC, United States

**Keywords:** transdermal biosensing, microfabrication, microfluidics, microneedle, luminescent sensors, fluorescent biosensors

## Abstract

The interaction of sensing components with body fluids is a basic requirement for clinical diagnostics; a variety of novel platforms have recently been developed for invasive and non-invasive sensing. In this manuscript, recent advancements related to minimally invasive platform for biosensing are reviewed. Many approaches have been utilized for generating minimally invasive platforms that require a small volume of body fluid; for example, the use of small-scale needles known as microneedles for minimally invasive detection has been demonstrated. The use of capillary action in microneedle-assisted biosensing may facilitate the detection of analytes in body fluids. This review considers recent innovations in the structure and performance of minimally invasive sensos.

## Introduction

The development of biotelemetry instruments for monitoring the physiologic activity (e.g., EKG, heart rate, respiratory rate, and oxygenation rate) of astronauts in the Apollo in the United States space program led to the development of several successful devices for assessing and altering physiologic activities of hospitalized patients during the 1970’s (Hawkins and Zieglschmid, 1975). The growth in the number of individuals suffering from chronic health conditions (e.g., diabetes, hypertension, chronic heart failure) who would benefit from home-based monitoring is driving the growth in new biosensor technologies. Blood glucose, lactate, ethanol, electrolyte, cholesterol, creatinine, urea, glutamate, and neurotransmitters are the most clinically significant analytes; biosensors for these analytes can enable remote patient monitoring and care. Sensors for these chemicals require the interaction of the sensing components with body fluids, followed by subsequent amplification and signal display. At the present time, most *in vitro* blood glucose monitors (e.g., the MediSense^®^ device) require patients to obtain blood via a fingerprick to obtain a blood sample. The fingerstick procedure can become stressful and painful when repeated several times over the course of a day. The preferred mode of sensing involves the use of biological components that act as selective recognition elements in combination with physio-chemical transducers to generate clinically significant data. Most successful analytical information is recorded with whole blood samples and involves an invasive approach to access an appropriate volume of blood. Recent research efforts have involved detecting chemicals in sweat, saliva, breath using wearable sensors, which contain skin-compatible materials and include miniaturized flexible/stretchable electronic systems, wireless communication modules, and electrochemical biosensors. These devices often contain soft hydrogels and other low-modulus materials for conformal interaction with the epidermal layer of the skin. These devices are intended for ambulatory use outside a clinical laboratory environment, including miniaturized components that are capable of real time extraction, capture, and analysis of biochemicals (Canovas et al., 2019). This review considers technologies associated with biosensing of body fluids that are extracted either through invasive or minimally invasive mechanisms. Considerable work has been done on exploring minimally invasive platforms, including: (a) acquisition of body fluid through capillary action and (b) use of microneedles for transdermal sensing. The role of minimally invasive platforms in biosensing is considered, including (i) biosensor technology, (ii) commercial innovation in biosensor technology, and (iii) the transition from invasive to minimally invasive platforms. The sample volume is the function of reaction area of the biosensing component; as such, it is important to understand the structure and operation of the technology that serves as the basis for the function of the biosensor.

### Biosensor Technology

**FIGURE 1:**
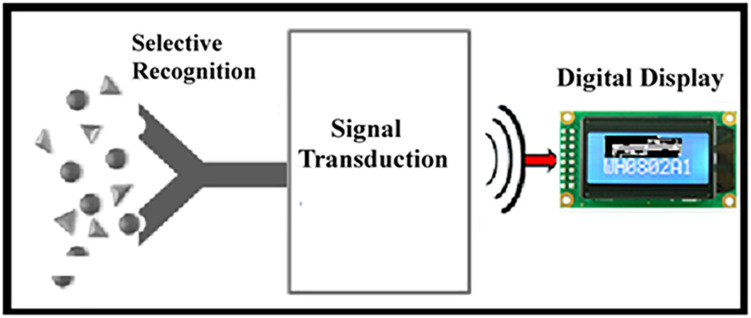
Schematic diagram showing the main components of a biosensor. A “selective recognition” event allows specific interaction of the targeted analyte with the biocatalyst, resulting in a change in the sensing component. This process is followed by “signal transduction” into a “digital display” as a function of analyte concentration.

Biosensors have recently emerged as economical devices for both *in vitro* and *in vivo* medical diagnostics; the development of new selective sensing mechanisms remains an active field of research Unlike more established and traditional branches of scientific inquiry, the development and testing of biosensors requires the use of several core disciplines. The interdisciplinary nature of biosensor development makes it difficult for researchers with different specialties to communicate well with one another or even agree upon an area of focus. Biosensors are generally considered to be devices that employ a biological sensing element as a recognition element; this recognition element is integrated within a transducer in order to translate the recognition event of a biological sensing element into a measurable signal. The biological elements can include active proteins, nucleic acids, receptors, or whole cells. These biological elements are selected for their highly specialized interactions with targeted analytes. For example, antibodies have specific binding sites, which selectively bind with a particular antigen. This process changes some physical parameter to produce an analytical parameter that is translated by the transducing element. Accordingly, the profile of a biosensor involves the combination of two major events, namely (i) the recognition event and (ii) the transduction event as shown in Figure 1. The nature and dynamics of the recognition event are a function of biological selectivity; the transduction event utilizes electrical engineering to generate a quantitative measurement of the recognition event via one of several mechanisms of signal transduction and amplification, namely (i) optical, (ii) thermal, (iii) electrochemical, (iv) mass change, and (v) a hybrid effort of the previously mentioned amplification technologies.

#### Biological Recognition Event

Selectivity is one of the requirements of analytical techniques that has severely restricted the practical implementation and subsequent commercialization of research on chemical sensors. Biological components represent sources of selectivity. In turn, a biosensor must meet the necessary requirement of selectivity. Several biological components that are currently utilized for biosensing are described in the following sections.

##### Enzyme

Enzymes are traditionally discussed in terms of their ability to accelerate or inhibit biochemical reactions. Enzymes catalyze reactions by very specific binding with a particular analyte (i.e., activating molecule), transforming it into a different biomolecular product (or products). In some cases, the activating analyte can cause a change in the enzyme, which induces a conformational change in the enzyme structure. The selectivity in enzyme-substrate interaction is possible as a result of the dynamics between the protein with respective substrate. Oxidoreductases enzymes that catalyze oxidation/reduction reactions have been explored in electrochemical biosensor design. For example, oxidase and peroxidase enzymes been utilized in commercialized glucose oxidase (GOD)-based biosensors (Grimes et al., 2006).

##### Immuno-receptor

Antibodies exhibit highly specific binding with other molecules (North, 1985). Elicited by organisms in response to an immune challenge, antibodies react to specific molecules that are considered “foreign,” which are known as antigens. Since an organism may come in contact with a large number of potentially harmful substances, a correspondingly large number of antibodies can be generated. Like enzymes, antibodies may be engineered or tagged to allow for monitoring of binding and consequential changes in conformation. One of the benefits of using antibodies is that the structure of antibodies is well-understood, which allows for thorough understanding of sensing events that involve antibody-antigen interactions.

##### Whole cell

Whole cell-based sensing is a well-established approach with numerous uses. Specialized cells are frequently employed; for example, sensory cells that contain specific chemoreceptors or neurons that detect specific neurotransmitter molecules are commonly used for whole cell-based sensing (Hazama et al., 1998). These interactions usually produce a form of electrical charge or action potential as a result of cell-molecule interaction, which can then be monitored to give an indication of the target molecule concentration. For instance, specifically selected cells that are sensitive to changes in glucose and glibenclamide have been used to monitor insulin secretion of other cells; signaling involves a series of current spikes that can be recorded through patch clamp techniques (Hazama et al., 1998). Other types of intact cells have also been used in biosensors; these studies investigate changes in metabolic activity or transmembrane potential that result from changes in the function of organelles, cellular proteins, and membrane receptors, or other cellular components. However, the true potential of cell-based biosensing lies in monitoring the many types of analytes using single whole cells. While the transduction of a multianalyte cell detection process is not straightforward, carefully selected or engineered cells are ideal for such an application as they can contain many different types of proteins and an equally large quantity of precise binding sites. In contrast to other proteins used in biosensing applications, proteins contained within cells do not suffer from the steric hindrance or denaturation that may accompany direct immobilization of the protein on the sensor surface. Unlike antibodies or enzymes that must be carefully harvested and separated for use in biosensors, a cellular biosensor would be self- replicating, enabling a reduction in the manufacturing cost and the development of a potentially reusable product.

#### Transduction Element

After the biological sensing component selectively interacts with the targeted analyte, a change in the physical parameters of the sensor occurs; a transduction process must be incorporated to collect data as a function of this physical change for quantitative detection of analyte-sensor interactions. Four approaches, namely (i) optical (Fernandes, 1998; Schult et al., 1999; Clapp et al., 2004; Rizzo et al., 2004; Eldamak and Fear, 2018; Feng et al., 2020), (ii) thermal (Zhang and Tadigadapa, 2004), (iii) mass (Zhou et al., 2002), and (iv) electrochemical, and serve as transduction elements for biosensing (Clark and Lyons, 1962). For example, fluorescent indicators can be excited and amplified with specific wavelengths of light. Under similar circumstances, one protein labeled with a particular fluorophore may emit light more brightly, while another fluorophore may remain unchanged. Due to the nature of resonant energy, when the two materials come into close contact (e.g., during a specific binding reaction in a biosensor), the energy can be redistributed between the two, causing the initially unaffected fluorophore to begin emitting more light. Forster resonant energy transfer (FRET) utilizes this phenomenon to examine transferable resonant energy before and during biochemical reactions between various biological materials (Clapp et al., 2004; Rizzo et al., 2004). The unique properties of quantum dots (e.g., the ability to emit light at different wavelengths and the ability to act in certain instances as semiconductors) have made these materials ideal for use in FRET analysis. Quantum dots offer higher emission and a larger range of distinct emission wavelengths than other fluorophore tags. The accuracy of FRET transduction is directly related to the extent of overlap of the spectra of the fluorescently-labeled materials (Clapp et al., 2004). The FRET technique is currently constrained by a limited selection of fluorescent tags (Rizzo et al., 2004). In one recent study, a cyan fluorophore was altered to improve the quantum yield and the extinction coefficient; as a result, the modified materials may provide more accurate data with less noise (Rizzo et al., 2004). Research is underway to develop a more extensive array of fluorescent tags with appropriate characteristics for FRET detection. Multianalyte analysis based on FRET technology could prove extremely useful for biosensing of multiple biological parameters in a given patient (Rizzo et al., 2004).

### Commercial Innovations in Biosensor Technology

It is important to consider mechanisms that allow biological recognition events to be converted into detectable signals. These mechanisms must be integrated into manufacturing processes for commercial translation. In addition, manufacturing costs as well as instrument errors must be minimized. While the wide variety of available biosensor technologies allows for innovation of new fabrication techniques, it does not lend itself to standardization of a particular protocol across biosensor manufacturer. Many biosensors have been successfully developed; however, the transition from basic research to release of a marketable product may be simplified by evaluating the manufacturing process. Two important stages determine the commercial viability of a biosensor: (a) the technology of the manufacturing process, which is an important parameter for minimizing cost and error; and (b) a user friendly design, with the possibility of transition toward a non-invasive approach.

#### Biosensor Manufacturing Process

##### Lithography and stereolithography

Lithography allows for the systematic construction of biosensor components through the superposition of layers of material. Chemical or other treatments may be applied to selected areas of the layered substrate. The various forms of lithography generally provide a high degree of precision in the fabrication of biological sensing components; however, there are limitations in terms of the types of materials and geometries that can be processed using this approach.

Photolithography, stereolithography, and electron beam lithography are common techniques used in the fabrication of biosensors. Photolithography relies on the use of exposure of material to light (usually ultraviolet light) in order to build, remove, or change the properties of a given material. This particular manufacturing technique is flexible with regard to the desired characteristics of the patterned material and use of the material in the end product. Stereolithography uses a laser or lamp to solidify a liquid polymer in a layer-by-layer manner in order to form a three-dimensional structure. Electron beam lithography involves the use of an electron beam to selectively etch away portions of a surface with a high dimensional precision. Many other adaptations of these techniques exist, including use of electrochemical methods that rely on the adsorption of protein onto a surface (Lai et al., 2006). All of these mechanisms relate to the addition and removal of material through the use of templates, chemical processes, and physical processes.

All of these micromanufacturing techniques are capable of preparing materials with small-scale features for biosensor applications. Stereolithography offers greater capabilities than photolithography in terms of the available geometries that can be produced; in addition, this technique allows for the manufacture of multiple biosensor components. However, if the geometry of the biosensor is too complex, support structures may be needed during the stereolithography manufacturing process. These support structures often need to be removed before the biosensor can be used; the support structure removal process adds to the manufacturing cost.

Given the versatility of lithographic procedures available to manufacturers, the inclusion of lithographic techniques in the biosensor manufacturing processes seems straightforward. Methods that are inexpensive and less labor intensive (e.g., photolithography and stereolithography) are more likely to become utilized in large-scale biosensor fabrication. Electron beam lithography may provide superior pattern quality at small dimensions; however, it cannot be implemented by manufacturers in a cost-effective manner for biosensor manufacturing.

##### Screen printing technology

One popular method of patterning materials is screen printing; for example, sensing materials or circuitry can be patterned using this approach. This method is inexpensive and straightforward, producing highly functional biosensors with small-scale feature sizes. A wire/cloth mesh is used to transfer ink onto a substrate (Licari and Enlow, 1998). A squeegee and flood bar are moved across the screen to fill the open mesh apertures with ink; the screen touches the substrate momentarily along the line of contact with a reverse stroke. This phenomenon causes the ink to wet the substrate and be pulled out of the mesh apertures when the screen springs back after the blade has passed. This phenomenon yields an impression on a mess stencil. Screen printing technology is currently used for the manufacture of electrodes and disposable printed electrodes. This mechanism has also been employed by researchers to print sensing materials on the transducer surface of a biosensor. Screen printing of biological materials on the surface of a printed electrode requires an active printing ink made from the homogenization of the biological material and a stabilizer. This mechanism can also be used to prepare circuitry. Electrically conductive or insulating inks are applied to a surface, which then dries and/or cools to form a continuously connected circuit pattern that functions as an electronic component.

The advantages of screen printing include low cost, rapid turnaround, and good gasketing (absence of smear). In the screen printing process, the screen is propped up by the emulsion location in the openings. The squeegee is moved down on the screen, enabling the screen to make contact with the substrate. The squeegee moves along the screen surface, pressing paste through the openings to cover the desired areas of the substrate. The process is dependent on the snap-off distance and the tension of the screen; these parameters determine how the screen peels out of the ink after the squeegee has gone by. The parameters that determine the quality of the screen printing process include the ink, printer, substrate, screen, and squeegee. The thickness, thickness uniformity, resolution, and number of voids can be modulated; for example, the screen mesh count controls the print thickness. Screen printed electrodes containing three microbands (e.g., working electrode, reference electrode, and counter electrode) have potential commercial viability.

##### Inkjet printing

Inkjet printing is a procedure for the specific patterning of any substance that can homogeneously be dispersed in a low viscosity solution in the form of an “ink.” The accuracy of inkjet drop placement depends largely on the properties of the ink, including the viscosity and the degree of heterogeneity. Inkjet printers have been used to print patterns of inorganic materials such as polymers, metals, and nanoparticles. More recent work has involved patterning of proteins and other biological materials by means of inkjet printing. A cartridge holds the desired solution and disperses it in a controlled manner onto the surface in the form of droplets via piezoelectric or thermal actuation. This method does not require contact between the inkjet printer nozzle and the surface, which limits contamination.

Two primary types of inkjet printing mechanisms have been utilized for the production of biosensors, piezoelectric and thermal printers. In piezoelectric nozzles, a piezoelectric material vibrates when a voltage is applied. The mechanical movement of the piezoelectric material creates pressure gradients, which lead to the movement of fluid either from the cartridge to the nozzle or from the nozzle to the surface. The ink is expelled from the nozzle with application of pressure; no change in temperature is associated with this process. The thermal printing mechanism utilizes applied voltage waveform to activate a heat source, which vaporizes the ink into a bubble. This process in turn creates a pressure gradient when the bubble is released at the nozzle tip, pulling additional ink through the nozzle (Sen and Darabi, 2007).

The application of high temperatures associated with thermal inkjet printing or high pressures associated with piezoelectric inkjet printing is a cause for concern when patterning biological materials. It is known that an ink containing inorganic molecules may be altered due to changes in viscosity or premature curing at high temperatures. It has been suggested that the heating process has minimal effects due to stabilizing materials (e.g., glycerol) in the ink that limit damage to the biological materials during the printing process (Setti et al., 2004). Both mechanisms of printing provide accurate and reproducible patterning of biological materials. The use of inkjet printing to manufacture multianalyte arrays and other types of biosensors has been suggested by Carter et al. (2006).

#### User Friendly Design of a Commercial Biosensor

One factor that determines the commercial viability of a portable biosensor is that it must assume minimal or no expertise of the end user. In addition, the life span of the sensing component under environmental conditions associated with storage and use must be considered. Accordingly, the stability of biological components within the biosensor often determines the stability and lifespan of the biosensor.

The structural dynamics of biological component during the biosensing event governs signal generation (Figure 1). The simplest type sensing event may be understood from the use of the glucose oxidase enzyme for the detection of glucose levels. The development of portable electrochemical biosensors for blood glucose measurements has been highly impactful and commercially successful. In these devices, an enzyme-printed disposable electrode is precisely inserted at the appropriate location in a dedicated electronic meter with a digital display. The biochemical reaction takes place within a defined biocatalyst layer made from the membrane-forming component along with a stabilizer. The electron hopping sites within the membrane matrix and the overall impedance of the membrane matrix determine the charge transfer rate. Therefore, it is important to consider (i) the general mechanistic approach on electron exchange as a function of the enzymatic reaction; (ii) the effect of the thin film on the rate of signal transduction; and (iii) the sample volume requirement for biosensing.

##### General mechanistic approach on electron exchange as a function of the enzymatic reaction

**SCHEME 1:**
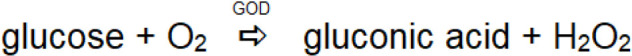
Reaction scheme for the first generation glucose biosensor.

In blood glucose sensing, the data is the function of the glucose oxidase-catalyzed reaction based on the reaction. The first-generation glucose biosensor based on the electrochemical mode of signal transduction relies on the natural enzymatic reaction glucose oxidase-catalyzed reaction, in which oxygen acts as an electron donor to regenerate the reduced glucose oxidase (Scheme 1). The reaction involves the selective interaction of blood glucose with glucose oxidase (GOD), converting glucose into gluconic acid. Glucose oxidase is reduced, followed by regeneration into the oxidized form by natural oxygen.

**SCHEME 2:**
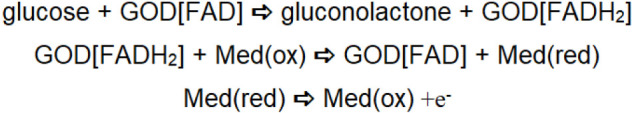
Reaction scheme for the second generation glucose biosensor.

Accordingly, the technology of blood glucose sensing based on this reaction scheme involves either the consumption of oxygen or the formation of hydrogen peroxide as a function of glucose oxidase-catalyzed reaction with blood glucose. The consumption of oxygen is probed through the use of an oxygen electrode. Based on this reaction scheme, the first commercial design of glucose sensor was launched by the Yellow Springs Instrument Company. On the other hand, hydrogen peroxide undergoes both reduction and oxidation at the electrode surface; analysis of hydrogen peroxide levels may also be used for glucose biosensing. The major problems associated with electroanalysis of hydrogen peroxide are: (i) poor sensitivity associated with the reduction of hydrogen peroxide, which takes place at a relatively low potential, and (ii) high overvoltage associated with the oxidation of hydrogen peroxide. In addition, the major problem associated with the enzymatic reaction as shown in Scheme 1 is the possibility of low oxygen tension at the site of the enzymatic reaction causing a rate-limiting effect, which in turn reduces the reliability of the glucose sensor. These issues led to the development of another reaction scheme that allows regeneration of the reduced enzyme in the absence of oxygen (Cass et al., 1984); this involves the use of an electron transfer mediator (Med.); the second generation of enzyme electrodes is shown in Scheme 2.

**SCHEME 3:**
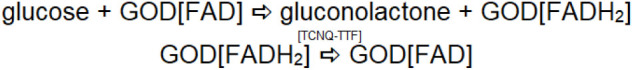
Reaction scheme for the third generation glucose biosensor.

Second generation glucose biosensors involve the participation of an oxidized form of the redox mediator at the site of the enzymatic reaction, which reacts with reduced glucose oxidase and converts it to a native form of enzyme, resulting in it being reduced. The reduced version of the redox mediator is regenerated electrochemically. During this process, the second order rate constant for the reaction between reduced glucose oxidase and the oxidized form of the redox mediator governs signal amplification and the overall response of the sensor. Accordingly, electron transfer mediators with appropriate second-order rate constants for transfer of electrons have been explored. Other criteria of redox mediators include insensitivity to changes in pH, temperature and ionic strength. Many ferrocene derivates show potential for effective regeneration of redox enzymes and glucometers based on this mechanism have been successfully commercialized. The use of redox mediators has been relatively reliable for single use applications (e.g., in commercially available blood glucometers). During subsequent measurements, these mediators leach out from the surface, affecting the rate of the enzyme-catalyzed reaction. These limitations led to consideration of the use of organic metals (Ferraris et al., 1973) such as tetracyanoquinodimethane (TCNQ) and tetrathiafulvalene (TTF), which can serve as efficient mediator’s for detecting the glucose oxidase-catalyzed reaction when incorporated within graphite paste (Pandey et al., 1991). The limitations mentioned above lead to the exploration of another artificial system for effective regeneration of the redox enzyme during the enzymatic reaction; another enzyme electrode concept was introduced. Third generation enzyme electrodes such as TCNQ and TTF, which show good redox activity, can serve as efficient mediators for regeneration of the reduced enzyme or cofactor. However, TCNQ is anionic in nature; TTF is cationic and allows charges transfer complexation when mixed together to form an organic metal. The organic metal acts as an efficient electrocatalyst (Lyons et al., 1993) and allows for regeneration of glucose oxidase as shown in Scheme 3. TCNQ-TTF acts as an efficient electrocatalyst for regeneration of the redox enzyme in this approach.

#### Effect of Thin Film on the Rate of Signal Transduction

Stabilization of the biocatalyst or enzyme at the surface of the electrode requires the presence of organic additives that exhibit the tendency to form a membrane matrix of defined porosity. A relatively porous matrix allows faster charge transport. On the other hand, it should also be noted that highly porous matrices are susceptible to leaching of the sensing components during electrochemical operation. The design of electrochemical biosensors based on mediated mechanisms also requires the presence of small redox molecules within the membrane matrix, which are more susceptible to leaching than the biocatalyst. Although disposable electrodes do not suffer from the limitation of leaching of the sensing components, they do depend on the close integration of the sensing components within the membrane matrix. Due to these challenges, disposable test strips are a commonly utilized approach for portable biosensing.

**FIGURE 2:**
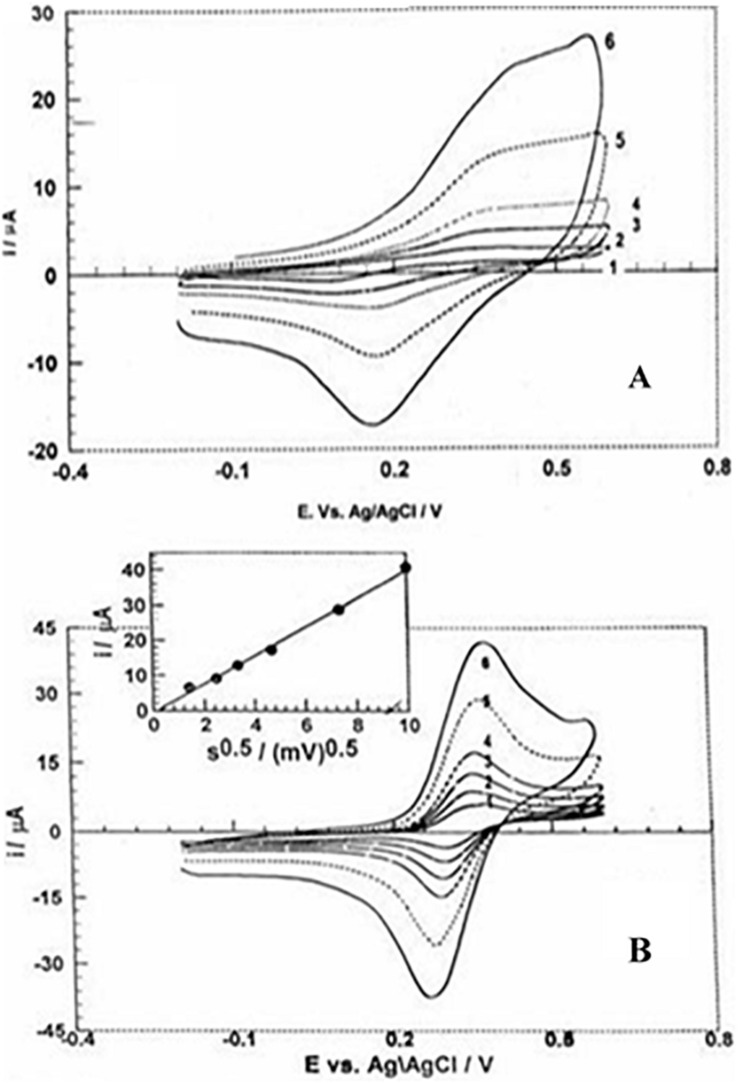
Cyclic voltammograms of ferrocene monocarboxylic acid at different scan rates within an organically modified silicate electrode made without **(A)** and with **(B)** -Pd-C- and -Pd-Si- linked alkoxysilanes.

Improvements to biosensor technology also require innovations in membrane matrix technology. One innovation in third-generation enzyme electrode involved the development of a hydrophobic organic metal layer, which itself functions as an immobilization support. The role of nanostructure domains in the membrane matrix has been examined (Pandey et al., 1999a). Our findings on the use organically modified silicate (Ormosil) materials demonstrated that Ormosils served as an efficient biocompatible membrane matrix for biosensing applications in which both a biocatalyst and a redox mediator may be retained for relatively long time while retaining biological activity of the biocatalyst and stability of the small redox mediators (Pandey et al., 1999b). However, when these sensing elements (i.e., biocatalyst and redox mediators) are encapsulated in the nanostructured matrix, the charge transport is sluggish and the redox mediator is not able to communicate with active site of biocatalyst as required for mediated electron exchange (a criterion of second generation enzyme based electrochemical biosensors). A nanostructure matrix of an organically modified silicate can be made by the specific interaction of two functional alkoxysilanes. For instance, trimethoxysilane and 3-glycidoxypropyltrimethoxysilane yielded a biocompatible membrane matrix for encapsulating both glucose oxidase and ferrocene monocarboxylic acid (Pandey et al., 1999a, b). Sensing components encapsulated in an organically modified silicate film on the electrode surface exhibit biochemical interactions due to the restricted mobility of the redox mediator within the nanostructured matrix. As electrocatalyst such as palladium may be incorporated within the nanostructure domain; a study of functional alkoxysilane-mediated formation of palladium nanoparticles (Pandey et al., 2001). Incorporation of biocatalysts and a redox mediator (e.g., ferrocene monocarboxylic acid) within a nanostructured matrix made from palladium-linked organosilanes provided better results than a homogeneous solution; the as made nanostructured matrix behaved as solid solution. The electrochemistry results of the ferrocene monocarboxylic acid encapsulated ORMOSIL electrode are shown in Figure 2.

### Sample Volume During Biosensing

The transition from invasive to minimally invasive devices has been facilitated by the reduction in the sample volume that is required by new types of electrochemical biosensors. An electrochemical biosensor requires the interaction of finite amount of blood with the sensing component to a yield quantitative signal of the analyte concentration. This requirement at first glance appears to necessitate the use of an invasive technology for blood sampling. One challenge associated with self-monitoring of blood glucose levels is that the sample volume required for analysis depends on the reaction area of the sensor device. Approaches that require larger blood sample are less user friendly. Improvements in biosensor technology to reduce sample volume are a significant focus are in biosensor research. In most modern blood glucose sensors, a light prick on finger is required to extract small quantity of blood sample, which can be rapidly applied to the reaction area. Capillary action is used to facilitate transfer of the blood to the sensing area of enzyme electrode. Under 2 μl of blood is required for a measurement. Current blood acquisition approaches (e.g., lancets)are associated with the generation of wounds and the potential risk of infection.

Efforts are underway to develop minimally invasive and less painful approaches for patient self-monitoring with biosensors, including the use of wearable sensors. Wearable sensors are point of care biosensors, allowing for minimally invasive monitoring of physiological functions and elimination of fluid transfer between subject and device; these devices are capable of providing real-time analysis of the user’s biochemical condition (Windmiller and Wang, 2013; Kassal et al., 2015; Choi et al., 2018). Novel epidermal electronic devices consisting of printed flexible circuits that can be stretched and bent to mimic skin elasticity have been fabricated for performing electrophysiological measurements such as measuring temperature and hydration as well as monitoring electrical signals from brain and muscle activity (Kim et al., 2011). Wearable sensors in the form of temporary tattoos with screen printed electrodes have been developed to attach directly to the skin for detection of lactate levels in sweat (Jia et al., 2013). However, many body metabolites do not appear in the same levels in sweat as in other body fluids. As such, efforts are underway point of care diagnostics that are minimally invasive and result in real time biosensing with high sensitivity. The use of microneedles, miniature lancet-, thorn-, or hypodermic needle-shaped devices with heights below 1 mm, for minimally invasive analytical systems has been considered. The devices are capable of acquiring biological fluids such as interstitial fluid in a minimally invasive manner due to their ability to puncture the stratum corneum layer of skin while not interacting with deeper layers of the skin, which contains tissues that are associated with pain, blood flow, and sensation (El-Laboudi et al., 2013; Miller et al., 2014).

#### Microneedle-Based Glucose Monitoring

A major focus of microneedle-based sensor research involves microneedle-based monitoring of glucose levels. For example, Zimmermann et al. (2003) demonstrated passive diffusion of interstitial fluid from a human finger with a hollow microneedle device; glucose levels were detected using enzymatic electrochemical electrodes, which were located beneath the microneedle array. Interstitial fluid was wicked through eight 200 μm tall microneedles and mixed with a buffer solution. The glucose biosensor showed a readily discernible signal at the beginning of testing; however, the sensor responsiveness was not sustained over the course of the study. The limitations of the device were associated with the fact that device function relied on movement of a small amount of interstitial fluid through passages within the device.

**FIGURE 3:**
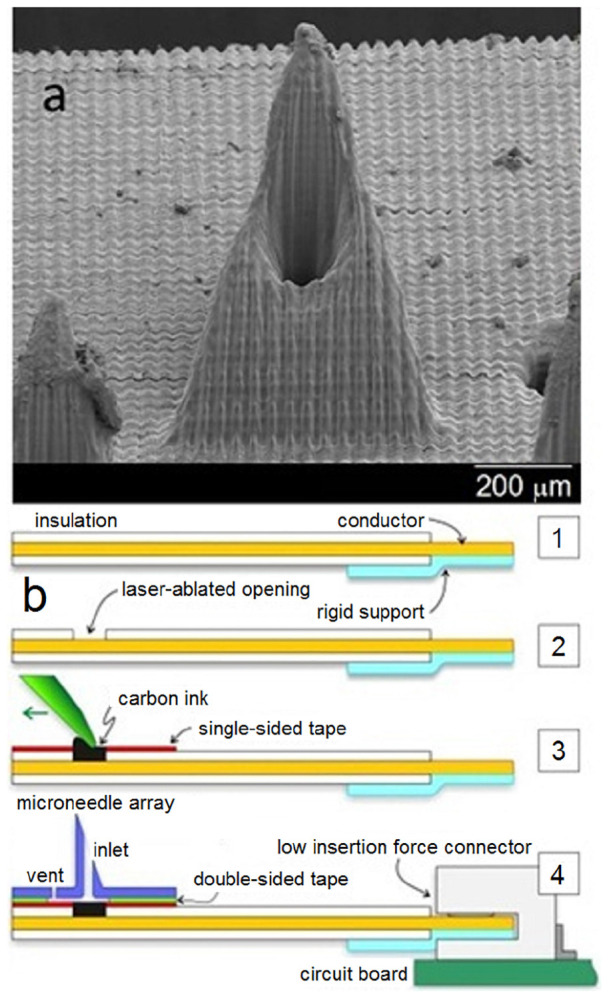
Scanning electron micrograph of a single microneedle **(a)** and schematic of the microneedle-biosensor assembly **(b)**. Reprinted with permission from Miller et al. (2012).

An amperometric glucose sensing smart patch was recently described that used a conducting polymer, poly (3, 4-ethylenedioxythiophene) (PEDOT), to entrap glucose oxidase directly on the surfaces of solid stainless steel microneedle arrays. PEDOT provided a biocompatible environment to trap the active enzyme; it allowed glucose to diffuse into the polymer matrix. In addition, its electrical properties provided a low voltage signal transduction pathway. A particular advantage of this technique is the ability of the microneedles to sample directly from interstitial fluid without complicated microfluidic components and separated sensor architectures. The microneedle-based sensors were calibrated and performed within the physiological range of glucose. However, the microneedle-based sensors were tested outside of the cells; as such, it is currently unknown if shearing of the soft polymer coating on the exterior of the microneedle can occur during skin penetration. Miller et al. (2012, 2016) and Wang et al. (2017) reported on the design of a microneedle-based sensor (Figure 3), which involves packing modified rhodium-doped carbon paste into open wells on an insulated wire strip for the simultaneous multiplexed detection of glucose, lactate, and pH. Carbon dioxide laser was used to ablate small holes to expose copper in a flat flexible cable. Each hole was packed with carbon paste formulations that were tailored specifically for glucose, lactate, or pH detection. This sort of multiplexed microneedle-based sensor is useful since abnormalities in the levels of the three biomarkers can indicate physiologic changes associated with metabolic acidosis as well as other acute or chronic medical conditions.

The fabrication of a screen-printed electrode (SPE) and a microneedle-assembled electrochemical sensor for transdermal biosensing was recently reported (Pandey et al., 2018). Polyethylenime-mediated Prussian blue nanoparticles and gold nanoparticles were made as previously disclosed. Gold nanoparticles were then mixed with Prussian blue nanoparticles; the desired amount of a homogeneous suspension of polyethylenimine-modified Prussian blue-gold nanohybrids-enzyme ink was layered over a freshly made SPE followed by curing of the same. The modified SPE was joined to a microneedle assembly. The microneedle-based transdermal biosensor for on-chip electrochemical biosensing can be fixed on the skin with a simple bandage. These PBNP-modified SPEs may be used for electrochemical sensing of hydrogen peroxide.

**FIGURE 4:**
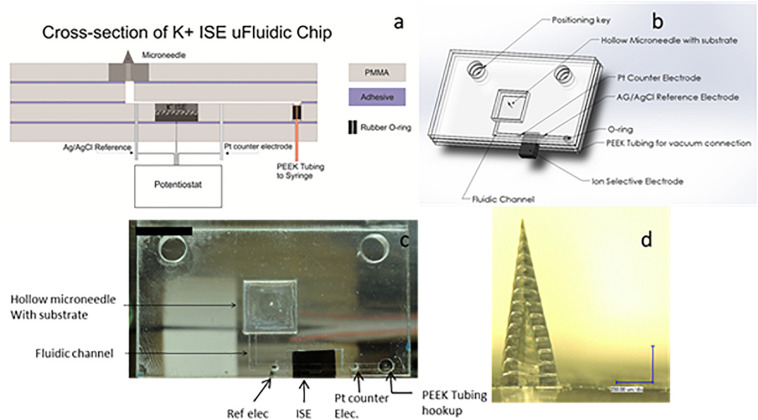
**(a)** CorelDraw rendering of a cross-section of the K + ion-sensitive electrode microfluidic chip. **(b)** Solidworks drawing of the K + ion-sensitive electrode microfluidic chip. **(c)** Image of the microfluidic chip with on chip reference and counter electrodes. **(d)** Optical image of a single hollow microneedle made with two-photon lithography (two-photon polymerization) (scale bar = 250 μm). Reprinted with permission from Miller et al. (2014).

A transdermal sensing device designed to measure physiologically relevant concentrations of potassium ions has also been described (Figure 4). In this device, a porous carbon electrode was evaluated as a transducer for an ion-selective electrode (Miller et al., 2014). The porous carbon ion-selective electrode was used for detection of potassium ion levels, and exhibited a detection range from 10^–5^ to 10^–2^M with a near Nernstian slope of 57.9 mV per decade and rapid stabilization (≈20 s). The porous carbon ion-selective electrodes showed no response to interfering sodium ions. The solid-state ion-selective electrode was incorporated into a fluidic chip along with a hollow microneedle. The method allows for a hollow microneedle to draw fluid over a three-electrode system within a microfluidic chip. This approach provides an attractive platform for an on-body sensing system for monitoring potassium; it can easily be expanded to detect a host of relevant physiological markers in a point of care diagnostic device.

#### Microneedle-Based Drug Delivery

In addition to sensing, microneedles can be used for local drug delivery. For example, Singh et al. noted that microneedles may be useful for localized drug delivery ocular tissue, including delivery to the anterior and posterior segment of the eye (Thakur Singh et al., 2017). Metallic microneedles offer a robust platform for minimally invasive drug delivery and biosensing applications (Cahill et al., 2018). Microneedles can also be used for cell delivery; for example, these devices can be used for cell delivery for treatment of vitiligo (Gualeni et al., 2018). A recent review summarizes some of the key challenges faced by microneedles for use in sensors and other wearable devices (Madden et al., 2020).

#### Comparison of Invasive, Minimally Invasive, and Non-invasive Sensors

Technological advances in terms of miniaturization of chemical sensors have revolutionized the clinical laboratory work and are now enabling continuous and instantaneous patient monitoring via minimally invasive devices. The sensing event requires close contact of the targeted analyte with the recognition element of the sensor, followed by signal transduction and communication of the result. Several sensors are entirely non-invasive, including physical sensors such as temperature sensors. Non-invasive wearable chemical sensors (e.g., a tattoo containing an electrode that is coated with a sensing element) can detect analytes that are present in sweat. Invasive technologies typically involve chemical analysis of blood or other body fluids by the sensor. The transition from invasive to minimally invasive sensing technologies for point of care patient monitoring has been enabled by a reduction in the minimum sample volume that is required for sensing (Pandey et al., 2019).

The form factor for the device can be optimized for use by infants, individuals with disabilities, or older individuals. For example, a pacifier biosensor has been demonstrated, which enables wireless and non-invasive chemical monitoring of an infant’s saliva (García-Carmona et al., 2019). Electrochemical detection of tetrahydrocannabinol in saliva has also been demonstrated (Stevenson et al., 2019). Non-invasive sensing of breath may be used for detection of biomarkers linked to lung cancer, oxidative stress, diabetes, and other diseases; a recent review summarizes advances in the field of electrochemical enzymatic breath biosensors (Gaffney et al., 2020).

## Conclusion

This review describes the efforts on biosensor design for medical applications with special attention to the role of minimally invasive platforms. Efforts to minimize the sample size for biosensing through capillary action were described. Recent innovations in minimally invasive technology based on transdermal biosensing were demonstrated. Combination approaches offer great benefits for enhancing the sensitivity of portable sensors; for example, the combination of an enzymatic assay with an optofluidics sensing system for vancomycin detection offers high sensitivity and a low detection limit in clinically relevant samples at low volumes. Hollow microneedles also provide significant benefits for portable sensors. For example, a device containing a hollow microneedle was developed, which draws fluid into a three-electrode system within a microfluidic chip; this approach provides an attractive platform for an on-body sensing system for monitoring potassium and can easily be expanded to other relevant physiological markers for next generation point of care diagnostic devices. These results suggest the potential of integration of sensors in portable devices for therapeutic drug monitoring and other biomedical applications.

## Author Contributions

All authors assisted in preparation and review of the manuscript.

## Conflict of Interest

The authors declare that the research was conducted in the absence of any commercial or financial relationships that could be construed as a potential conflict of interest.
